# Reference values for body composition and associations with blood pressure in Kenyan adults aged ≥50 years old

**DOI:** 10.1038/s41430-018-0177-z

**Published:** 2018-05-15

**Authors:** Madeleine C. Bastawrous, Carmen Piernas, Andrew Bastawrous, Jason Oke, Daniel Lasserson, Wanjiku Mathenge, Matthew J. Burton, Susan A. Jebb, Hannah Kuper

**Affiliations:** 1 Nuffield Department of Primary Care Health Sciences, University of Oxford, Oxford, UK; 2 International Centre foer Eye Health, Clinical Research Department, London School of Hygiene & Tropical Medicine, London, UK; 3 Department of Geratology, Oxford University Hospitals NHS Foundation Trust, Oxford, UK; 4 Rwanda International Institute of Ophthalmology, Kigali, Rwanda; 5 The Fred Hollows Foundation, Nairobi, Kenya; 6 Moorfields Eye Hospital, London, UK

**Keywords:** Risk factors, Epidemiology

## Abstract

**Background/objectives:**

To develop age- and sex-specific centile reference curves for fat-free mass (FFM) and fat mass (FM) adjusted for height in an adult Kenyan population and to investigate the association between FM, FFM and blood pressure (BP).

**Subjects/methods:**

Measures of body composition from bioimpedance analyses and BP were collected in 1995 participants aged ≥50 years in Nakuru County, Kenya. Reference curves were produced using the LMS method. Multivariable linear regression models were used to test the cross-sectional association between body composition indexes and BP.

**Results:**

The age- and sex-specific reference curves for body composition (FMI and FFMI) confirmed that FFMI is lower in both men and women with increasing age. FMI declines with age in women while among men the decline starts after 70 years. FFM was higher in men (47.4 ± 7.2 kg) than in women (38.8 ± 5.5 kg), while FM was lower in men (17.3 ± 8.1 kg) than in women (24.4 ± 10.2 kg). FMI, FFMI and BMI were all positively associated with systolic and diastolic BP, and after adjusting for body weight, FFMI remained positively associated with systolic BP and the FMI remained positively associated with diastolic BP. There was no evidence to suggest that FMI and FFMI were superior to measurement of BMI alone.

**Conclusions:**

These body composition reference curves provide normative data on body composition for older adults in Kenya. Further research should consider the prospective associations with health, including frailty-related outcomes.

## Introduction

There is growing interest in the effect of body composition, beyond body weight, on health outcomes linked to aging [1, 2]. Traditionally, nutritional assessment has relied upon the body mass index (BMI) (weight/height^2^), which is easy, cheap and convenient to measure, but it is limited because the relative proportion of fat mass (FM) and fat free-mass (FFM) is unknown. Changes in body weight may be due to changes in FM, FFM or both. Although there is a positive correlation between FFM and body weight within populations, FFM cannot always be accurately predicted from weight or BMI at an individual-level [2], and changes in BMI may obscure underlying shifts in the proportion of FM and FFM.

Bioelectrical impedance analysis (BIA) is a simple, portable, inexpensive and non-invasive technique to assess FFM and FM [3]; however, the use of body composition measurements in routine health surveillance and in clinical practice is limited by the lack of reference data to allow the classification of individuals at risk across the age spectrum. Centile charts have been developed to describe changes in FM and FFM throughout childhood [4, 5] and for adults in high- and middle-income countries [6–9]. However, body composition varies by population [10, 11], and loss of FFM may be related to ethnicity, diet, physical inactivity or disease in older adults [12, 13]. Reference data are especially limited in African populations [12] and resource-poor settings [14]. Population-specific body composition reference data will help to elucidate age-related changes associated with ill-health, and will allow more accurate characterization of individuals at greatest risk of disability and morbidity so they can receive targeted support. This is especially important in low- and middle-income countries where the double burden of non-communicable diseases (NCDs) and infectious disease coupled with limited health systems means that healthcare resources need to be carefully prioritized [15–18].

There is growing evidence that FM and FFM are independent risk factors for NCDs. FM has been shown to be strongly associated with increased risk of chronic diseases such as hypertension, hyperlipidemia and insulin resistance [19]. Conversely, reductions in FFM are characteristic of aging and independently associated with worse health outcomes and disabilities [20, 21], leading to frailty, reduced life expectancy in healthy subjects and impaired quality of life associated with weakness, alongside increased personal and healthcare costs [22–24]. In addition, loss of FFM, or more specifically skeletal muscle mass (SMM) in combination with increased adiposity, known as sarcopenic obesity, is increasingly recognized as a major health concern in the aging population because of its association with a higher risk of cardiometabolic and functional abnormalities [25]. A previous study found an increased risk of hypertension among those with sarcopenic obesity [26].

This study aimed firstly to develop age- and sex-specific centile reference curves for FFM and FM derived from BIA in an adult Kenyan population. Secondly, we aimed to investigate the association between demographic characteristics and body composition measures; and to compare the predictive value of FFM and FM relative to BMI for the association with systolic and diastolic blood pressure (BP).

## Methods

### Study population

This study used data from a subsample of participants (*n* = 1995) from the longitudinal Nakuru Eye Disease Cohort Study [27]. The primary aim of the study was to establish a survey which formed the baseline to a 6-year prospective cohort study to estimate the incidence and progression of eye disease in this population and the data collection reflected these goals. The initial study population was determined following a baseline population-based survey conducted in 2007–2008 with the aim of recruiting a sample of ~5000 participants aged ≥50 years, based on an expected prevalence of a visual acuity of <6/12 in the better eye caused by posterior segment disease of 3.0%. A total of 100 clusters each of 50 participants were selected with a probability proportional to the size of the population across Nakuru district. Households were selected within clusters using a modified compact segment sampling method. In total, 4381 participants at baseline (87.4% response rate) underwent complete examination across 100 clusters. A follow-up examination was conducted 5.6 years later (January 2013 to March 2014), in which 2170 (50%) participants were followed up. Among these, 1995 participants (*n* = 950 men and *n* = 1045 women, 92% of the follow-up sample) had complete data on body composition and BP, which was used for the present analysis.

Further details of all the procedures undertaken for all participants who attended an examination clinic at baseline and follow-up are available elsewhere [27]. Structured interviews were performed in the participant’s preferred language covering (i) demographic; (ii) past medical and ocular history including medication and relevant family history; (iii) known NCD risk factors; and (iv) socioeconomic status based on job, housing conditions, ownership of material goods and livestock which is translated in to a score based on previous work in the same population [28].

The study adhered to the tenets of the Declaration of Helsinki and was approved by the Ethics Committee of London School of Hygiene & Tropical Medicine (LSHTM Ref 6192) and by the African Medical and Research Foundation (AMREF) Ethics Committee (AMREF-ESRC P44/12).

### Anthropometry and BIA

As per protocol, a nurse recorded measures for height (m) (Leicester Height Measure, Chasmors Ltd, London) and weight (kg) (Seca 761, Williams Medical Supplies, London). BMI was calculated as the weight (kg) divided by the squared height (m). BIA was performed in non-fasting and resting conditions, using a Tanita Segmental Body Composition Monitor (BC-601, Tanita Corp). This study used data on FM and FFM, calculated as the sum of the predicted total muscle mass (which includes skeletal and smooth muscle and the water contained in those) and bone mineral mass.

Consistent with previous studies [9, 29, 30] and in order to account for the fact that people with the same body weight and FM percentage who differ in height will have different body composition status, height-adjusted indexes for body composition were calculated: FMI (fat mass/height^2^) and FFMI (fat-free mass/height^2^).

### Blood pressure

Three measurements of systolic and diastolic BP were taken using an Omron Digital Automatic Blood Pressure Monitor (HEM907, Omron) after the study participant had been seated for at least 10 min and each reading was taken 10 min apart. The mean of the three measurements was used.

### Development of centile curves

Centile curves were constructed for men and women separately using the LMS method, which summarizes the data in terms of three smooth age-specific curves, namely L (lambda), M (mu) and S (sigma) [31]. The M and S curves correspond to the median and coefficient of variation of each body composition measure at each age, whereas the L curve allows for the age-dependent skewness in the distribution of each body composition measure. The LMS function from the R package GAMLSS was used to estimate age-specific centile curves [32, 33]. Following the same format of other growth reference curves [4, 5], centile curves for FFMI and FMI were calculated, from the 2nd to the 98th percentiles, spaced two-thirds of an SD score apart.

### Statistical analyses

Stata version 14 was used for the statistical analyses. For descriptive analyses, means (SD) or samples (%) were used to describe demographic characteristics, anthropometric and health characteristics among men and women separately. Mean FMI and FFMI in each percentile were summarized for different age groups: 50–59, 60–69; 70–79; 80+. Four multivariable linear regression models were used to describe the population characteristics associated with each body composition index (i.e., FFMI and FMI) as well as for the BMI. Mean values were predicted for each demographic characteristic. The means presented are unconditional means calculated by averaging over the other variables in the model rather than estimating the means at fixed levels of the other covariates (reference levels). Four independent multivariable linear regression models were run to obtain beta coefficients (95% confidence interval (CI)) for the association between each measure (FFMI, FMI and BMI) and systolic and diastolic BP with adjustment for confounding variables such as age, gender, tribe, urban/rural, education, smoking and alcohol intake. In order to compare the predictive value between each body composition index (FFMI and FMI) and BMI, partial Pearson's correlation coefficients were obtained from the above multivariable models. Values of each body composition index and BMI were standardized into *z*-scores before running the models to allow comparison of their partial Pearson's correlation coefficients.

## Results

### Participant characteristics

From a total of 2170 Kenyan adults, a high proportion of participants had no education (17.1% men and 46.1% women) or only primary education (55.7% men and 31.8% women). The predominant tribal group was Kikuyu (Table 1).

**Table 1:** Demographic characteristics of the study population

	Men (*n*=950)		Women (*n*=1045)	
Demographic characteristics	Mean/n	SD/%	Mean/n	SD/%
Age (years)	67.8	8.7	66.6	8.9
Maximum education level				
None	162	17.1	482	46.1
Primary	525	55.3	409	39.1
Secondary/college/university	252	26.5	130	12.4
Missing	11	1.2	24	2.3
Socioeconomic status				
1 quartile (poorest)	244	25.7	220	21.1
2 quartile	194	20.4	228	21.8
3 quartile	186	19.6	241	23.1
4 quartile (richest)	187	19.7	183	17.5
Missing	139	14.6	173	16.6
Tribe				
Mixed	132	13.9	115	11.0
Kikuyu	559	58.8	702	67.2
Kalenjin	259	27.3	228	21.8

Mean BMI was within the healthy range for men: 23.1 ± 4.4 and in the overweight range for women: 26.1 ± 5.6 g/m^2^ (Table 2). The prevalence of obesity was lower in men (7.2%) compared to women (25.5%). FFM was higher in men (47.4 ± 7.2 kg) than in women (38.8 ± 5.5 kg), while FM was lower in men (17.3 ± 8.1 kg) than in women (24.4 ± 10.2 kg). Mean systolic BP for men was 134.0 ± 23.1 mm Hg and 130.8 ± 24.2 mm Hg for women. Mean diastolic BP was 82.7 ± 13.1 mm Hg for men and 82.6 ± 12.9 mm Hg for women. The majority of men and women never smoked (73.7% and 98.4%, respectively). A higher proportion of men consumed alcohol in the past or currently (55.7% and 25.3%, respectively) compared to a higher proportion of women who never consumed alcohol (60.1%).

**Table 2:** Health-related participant characteristics

	Men (*n*=950)		Women (*n*=1045)	
	Mean/*n*	SD/%	Mean/*n*	SD/%
Weight (kg)	64.7	13.5	63.2	14.8
Height (cm)	167.2	7.0	155.4	6.6
BMI (kg/m^2^)	23.1	4.4	26.1	5.6
BMI category				
Underweight (<20 kg/m^2^)	126	13.3	76	7.3
Normal weight (20–25 kg/m^2^)	530	55.8	400	38.3
Overweight (25–30 kg/m^2^)	226	23.8	303	29.0
Obese (>30 kg/m^2^)	68	7.2	266	25.5
FFM (kg)	47.4	7.2	38.8	5.5
FFMI (kg/m^2^)	16.9	2.1	16.1	1.9
FM (%)	25.6	7.7	37.1	8.2
FM (kg)	17.3	8.1	24.4	10.2
FMI (kg/m^2^)	6.2	2.9	10.1	4.1
FFM to FM index	3.4	1.8	1.9	1.0
Blood pressure				
Systolic (mm Hg)	134.0	23.1	130.8	24.2
Diastolic (mm Hg)	82.7	13.1	82.6	12.9
Smoking status				
Never	700	73.7	1,028	98.4
Former	125	13.2	3	0.3
Current	97	10.2	5	0.5
Alcohol consumption				
Never	167	17.6	628	60.1
Former	529	55.7	332	31.8
Current	240	25.3	65	6.2

### Body composition reference values

**Fig. 1:**
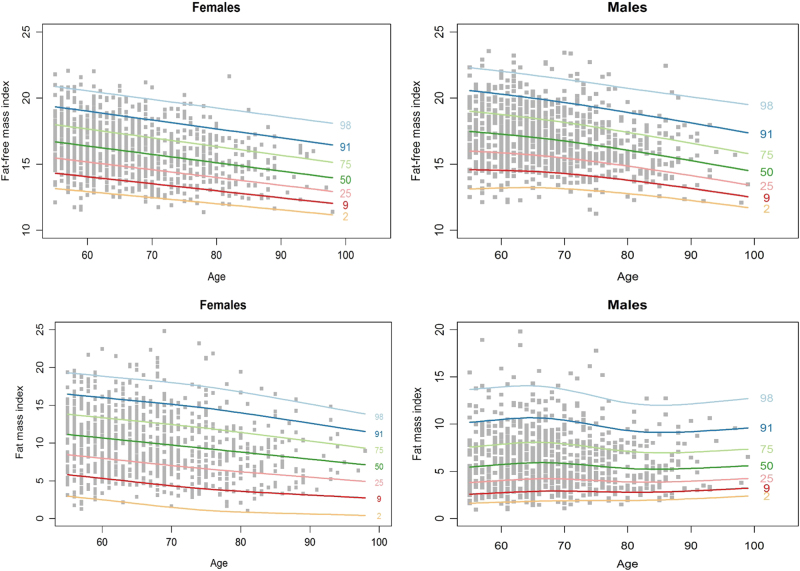
Age- and sex-specific centile reference curves for fat-free mass (FFM) and fat mass (FM) adjusted for height

Figure 1a–d shows age- and gender-specific centiles curves for FFMI and FMI. FFMI demonstrated a relatively consistent and significant rate of decline with age for both men and women (Fig. 1a, b). At the median, FFMI for men is 17.3 kg/m^2^ at 60 years and declines to 15.3 kg/m^2^ at 90 years (Supplemental Table 1). The younger women began with a lower FFMI than for men but followed a similar pattern with a median FFMI at 60 years of 16.4 kg/m^2^ declining to 14.5 kg/m^2^ at 90 years.

FMI for men peaked between 60 and 70 years, followed by a gentle decline before stabilizing between 80 and 90 years (Fig. 1c). In women, FM shows an on-going decline as age increases (Fig. 1d). The decline in men began at 60 years with a lower FMI of 5.7 kg/m^2^, decreasing to 5.3 kg/m^2^ at 90 years. Women however started at 60 years with a median FMI of 10.7 kg/m^2^ and reduced to 7.9 kgm^2^ at 90 years.

### Sociodemographic correlates of body composition

Multivariable linear regression models were used to investigate associations between each body composition measure and demographic and lifestyle characteristics (Table 3). Consistent with the results shown in the centile curves, mean FFMI was significantly lower in older age groups in both men and women, while FMI was significantly lower in older ages only among women. BMI was significantly lower in older age groups among men and women.

**Table 3:** Estimated marginal mean (SE)^a^ from descriptive models of participant characteristics in association with body composition status

	FFMI				FMI				FFM to FM				BMI			
	Men		Women		Men		Women		Men		Women		Men		Women	
	Mean	SE	Mean	SE	Mean	SE	Mean	SE	Mean	SE	Mean	SE	Mean	SE	Mean	SE
Age group																
60 (*n* = 1314 from 55–69 years)	17.3	0.1	16.3	0.1	6.3	0.1	10.3	0.2	3.4	0.1	1.9	0.0	23.5	0.2	26.7	0.2
70 (*n* = 466 from 70–79 years)	16.8	0.1*	15.9	0.1*	6.2	0.1	9.9	0.1*	3.4	0.1	1.9	0.0	23.0	0.1*	25.9	0.2*
80 (*n* = 188 from 70–79 years)	16.4	0.1*	15.5	0.1*	6.1	0.2	9.5	0.2*	3.3	0.1	2.0	0.1	22.5	0.3*	25.1	0.3*
90 (*n* = 27 from 70–79 years)	16.0	0.2*	15.1	0.2*	6.0	0.3	9.1	0.4*	3.2	0.2	2.0	0.1	22.0	0.4*	24.3	0.5*
Tribe																
Mixed (*n* = 247)	17.1	0.2	16.0	0.2	5.8	0.3	9.6	0.4	3.7	0.2	2.0	0.1	22.9	0.4	25.6	0.6
Kikuyu (*n* = 1261)	17.1	0.1	16.1	0.1	6.4	0.1*	10.2	0.1	3.3	0.1*	1.8	0.0	23.5	0.2	26.3	0.2
Kalenjin (*n* = 487)	16.6	0.1*	16.0	0.1	5.8	0.2	9.9	0.3	3.5	0.1	2.0	0.1	22.4	0.3	25.9	0.4
Residence																
Rural (*n* = 1506)	16.7	0.1	15.9	0.1	5.8	0.1	9.7	0.1	3.5	0.1	1.9	0.0	22.5	0.2	25.6	0.2
Urban (*n* = 489)	17.5	0.1*	16.5	0.1*	7.3	0.2*	11.3	0.3*	3.0	0.1*	1.9	0.1	24.8	0.3*	27.9	0.4*
Education																
None (*n* = 644)	16.5	0.2	15.8	0.1	6.1	0.2	9.3	0.2	3.4	0.2	2.0	0.0	22.6	0.4	25.1	0.3
Primary (*n* = 934)	16.9	0.1*	16.3	0.1*	6.0	0.1	10.5	0.2*	3.5	0.1	1.8	0.1*	22.9	0.2	26.8	0.3*
Secondary/college/university (*n* = 382)	17.4	0.1*	16.5	0.2*	6.6	0.2	11.5	0.4*	3.2	0.1	1.6	0.1*	24.0	0.3*	28.1	0.5*
Smoking																
Never (*n* = 1728)	17.1	0.1	16.1	0.1	6.4	0.1	10.1	0.1	3.3	0.1	1.9	0.0	23.5	0.2	26.2	0.2
Former (*n* = 128)	16.8	0.2	15.3	1.0	6.3	0.3	10.1	2.2	3.5	0.2	1.7	0.6	23.1	0.4	25.4	3.0
Current (*n* = 102)	15.6	0.2*	15.2	0.8	4.5	0.3*	8.6	1.7	4.1	0.2*	2.8	0.4*	20.2	0.4*	23.8	2.3
Alcohol intake																
Never (*n* = 795)	17.1	0.2	16.2	0.1	6.3	0.2	10.2	0.2	3.5	0.1	1.9	0.0	23.3	0.3	26.4	0.2
Former (*n* = 861)	17.0	0.1	16.0	0.1	6.2	0.1	10.1	0.2	3.3	0.1	1.9	0.1	23.2	0.2	26.0	0.3
Current (*n* = 305)	16.8	0.1	15.4	0.2*	6.0	0.2	8.6	0.5*	3.5	0.1	2.3	0.1*	22.7	0.3	24.0	0.7*

*P < 0.05

^a^ Results are estimated marginal means (SE) from multivariable linear regression models that included each body composition measure or BMI as the outcome and the demographic characteristics as predictors

Men and women from urban areas had a significantly higher FFMI, FMI and BMI than those from rural areas. Among participants with secondary education and above compared to those with no education, men had significantly higher FFMI and BMI, whereas women had significantly higher FFMI, FMI and BMI. Compared to never smokers, men who currently smoked had significantly lower FFMI, FMI and BMI. Among women, those currently consuming alcohol had a significantly lower FFMI, FMI and BMI compared to those who never consumed alcohol.

### Associations of body composition measures and BMI with BP

Table 4 shows standardized beta estimates and partial correlation coefficients for the association between each body composition measure and BP after adjustment for age, gender, tribe, urban/rural, education, smoking and alcohol intake. BMI, FFMI and FMI are all positively associated with both systolic and diastolic BP with correlation coefficients ranging from 0.13 to 0.22. After adjusting for body weight the associations were weakened. However, FFMI remained significantly positively associated with higher systolic BP and FMI with higher diastolic BP.

**Table 4:** Standardized beta coefficients and partial correlations^a^ relating each body mass measure with BP

	Systolic BP					Diastolic BP				
	*r*	Beta	95% CI		*P*	*r*	Beta	95% CI		*P*
Model 1^b^										
BMI	0.15	3.98	2.81	5.16	0.000	0.22	3.23	2.59	3.87	0.000
FFMI	0.15	3.64	2.55	4.74	0.000	0.18	2.39	1.79	2.99	0.000
FMI	0.13	3.22	2.13	4.31	0.000	0.21	2.86	2.26	3.45	0.000
Model 2^c^										
FFMI	0.07	2.61	1.03	4.18	0.001	0.04	0.68	−0.18	1.54	0.119
FMI	0.03	1.51	−0.61	3.63	0.162	0.06	1.60	0.45	2.75	0.006

^a^ Results are *r* (partial Pearson's correlation coefficient) and standardized beta coefficients (95% CI)

^b^ Model 1 adjust for age, gender, tribe, urban/rural, education, smoking and alcohol intake

^c^ Model 2 includes body weight (kg) plus all the other covariates included in model 1

## Discussion

This analysis presents novel age- and sex-specific reference curves for body composition (FMI and FFMI) in an older Kenyan population. This study confirms that FFM is lower in both men and women at older ages. FM also declines with age in women while among men the decline starts after 70 years. FMI, FFMI and BMI were all positively associated with systolic and diastolic BP, and after adjusting for body weight, the FFMI remained positively associated with systolic BP whereas the FMI remained positively associated with diastolic BP.

Most reference values for body composition are focused on predominately white populations from Sweden, Switzerland, the United Kingdom and the United States, with only a limited number of other non-white populations, including Japanese, African Americans and Mexican Americans [9, 11, 12, 14, 34, 35]. To our knowledge, there are no reference values for body composition specifically derived for Black African populations. The Japanese women have the lowest overall FFMI followed by similar FFMI for each age category from the Kenyan and Swedish populations; both the White and African American populations have the overall highest FFMI across the age categories. For men, the Kenyan population has lowest mean FFMI for each age category followed closely by the Japanese population. The male Swedish and both American populations have significantly higher FFMI for each age category. All ethnicities show a trend towards gradual decreasing FFMI for men, whereas those of United States and Sweden show a more static trend for women with a gradual decline in the Kenyan and Japanese populations. The overall lower FFMI and more rapid decline with age observed in the Kenyan cohort for both men and women compared to other studies may be due in part to poorer nutrition, particularly protein intake; between 2005 and 2007, dietary protein consumption for Kenya was 58 g/person/day and for United Kingdom, Sweden, United States and Japan was 104, 107, 114 and 92 g/person/day respectively [36]. Other potential explanations may include higher incidence of disease both infectious and non-communicable, and lower physical activity levels which may be related to comorbidity restriction. However, the differences in the trends observed highlights the need for ethnic-specific reference data on body composition.

The Swedish data show the population FMI to increase between 50 and 61 years and over 75 years [14]. In a slightly older Japanese population of women there was little change in FMI from 65 years to 85+ years, and this is in contrast to our Kenyan women aged over 55 years in whom FMI progressively declines. Among Swedish men, the FMI increases by 1 kg/m^2^ between the two age groups, whereas the Kenyan men exhibited a marginal decline in FMI with age and are more similar to the Japanese older male population who demonstrated a marginal decrease by 85+ years following a small increase at 75 years [11]. However, the sample size is small, limiting a more detailed interpretation.

Our second objective was to explore the relationship between FFMI and FMI with BP. Our models suggest both FFMI and FMI are positively associated with diastolic and systolic BP. However, there was no evidence to suggest that FMI and FFMI were superior to measurement of BMI alone. These findings are consistent with others studies that show that simple anthropometric measures such as BMI or waist circumference are strongly associated with obesity-related health risk factors such as high BP [37, 38]. BMI remains an inexpensive, easy to implement measure that is strongly associated with increased risk of components of the metabolic syndrome [39, 40] and thus an important screening tool.

The main strength of this study is the focus on older people in Kenya, while previous studies including African ethnicities have all included African Americans. We are unable to account for any bias that may have been imposed in using a follow-up subsample of the cohort, and generally those not included were those whom had died, moved away, were not located or were located but did not come to the examination. This may have affected the representativeness of the initial population sample. BIA was used to measure body composition because it is cheaper and easier to use in the field than reference techniques. BIA has been compared against reference methods such as whole-body dual energy X-ray absorptiometry showing high correlations [41–43], but it is less accurate at an individual level, especially at very high BMIs [43]. Moreover, the bioimpedance measures of body composition rely on predictive algorithms developed using data from diverse populations, mostly Caucasians. However, uncertainty remains over whether these estimates are less accurate for this Kenyan population [44]. Also, the reference values presented in this study are specific to the particular scale used.

An additional limitation is that we used measures of FFM rather than SMM. FFM includes SMM but also organ weight and other non-fat components such as bone mass, while SMM specifically is a more important tissue for the maintenance of glucose homeostasis and potentially a stronger biomarker for metabolic health [45]. While FFM and SMM are strongly correlated, we can only infer that the individuals in the lower centiles may have sarcopenia, a known risk factor for disability, independent of morbidity [1]. Nonetheless, this type of population-specific reference data for body composition in the elderly could potentially be used in health checks to identify individuals who may be at risk of sarcopenia, or to monitor changes in body composition over time to identify people with disproportionate losses of FFM, which may lead to frailty. Further research is needed into the relationship between FMI or FFMI and clinical outcomes in prospective cohorts, especially among people of non-Caucasian ethnicity given the known discrepancies between BMI and body composition across different ethnic groups [46] in order to identify clinical thresholds for intervention.

## Conclusion

This study presents novel reference data for FM and FFM in an older Kenyan population and confirmed that FFM is lower in both men and women with increasing age. It also shows a significant decline in FM in women with a smaller decline in men. FMI, FFMI and BMI were all positively associated with systolic and diastolic BP. Both FFMI and FMI show similar associations to those observed with BMI. The trends observed in the Kenyan population may be related to ethnic differences in body composition and highlights the need for ethnic-specific reference data on body composition. Further research should also consider the prospective associations with health, including frailty-related outcomes.

## Electronic supplementary material

Supplemental tables
