# Supplementary material for: Reference values for body composition and associations with blood pressure in Kenyan adults aged ≥50 years old
Source: Eur J Clin Nutr. 2018 May 15;73(4):558–65. doi: 10.1038/s41430-018-0177-z (PMC6124645; doi:10.1038/s41430-018-0177-z)
Supplement: Supplementary file 1 — Supplemental tables [file 41430_2018_177_MOESM1_ESM.docx]

Supplemental Tables

Supplemental table 1. Body composition centiles by age group and sex

|  |  | FFMI – Centiles | | | | | | |  | FMI - Centiles | | | | | | |  | FFM to FM - Centiles | | | | | | |
| --- | --- | --- | --- | --- | --- | --- | --- | --- | --- | --- | --- | --- | --- | --- | --- | --- | --- | --- | --- | --- | --- | --- | --- | --- |
| Sex | Age group | 2 | 9 | 25 | 50 | 75 | 91 | 98 |  | 2 | 9 | 25 | 50 | 75 | 91 | 98 |  | 2 | 9 | 25 | 50 | 75 | 91 | 98 |
| Men | 60 | 13.2 | 14.5 | 15.9 | 17.3 | 18.8 | 20.3 | 22 |  | 1.7 | 2.8 | 4 | 5.7 | 7.9 | 10.5 | 13.9 |  | 1.3 | 1.7 | 2.1 | 2.8 | 3.8 | 5.4 | 8.4 |
|  | 70 | 13.1 | 14.3 | 15.5 | 16.8 | 18.2 | 19.7 | 21.4 |  | 1.9 | 2.9 | 4.2 | 5.8 | 7.9 | 10.4 | 13.7 |  | 1.3 | 1.7 | 2.1 | 2.8 | 3.7 | 5.2 | 8 |
|  | 80 | 12.8 | 13.8 | 14.9 | 16.1 | 17.4 | 18.9 | 20.7 |  | 1.9 | 2.8 | 3.9 | 5.3 | 7.1 | 9.3 | 12.2 |  | 1.4 | 1.7 | 2.1 | 2.7 | 3.6 | 5.1 | 7.7 |
|  | 90 | 12.3 | 13.2 | 14.1 | 15.3 | 16.6 | 18.1 | 20.1 |  | 2.1 | 2.9 | 4 | 5.3 | 7 | 9.2 | 12.1 |  | 1.4 | 1.7 | 2.1 | 2.7 | 3.5 | 4.9 | 7.4 |
|  |  |  |  |  |  |  |  |  |  |  |  |  |  |  |  |  |  |  |  |  |  |  |  |  |
| Women | 60 | 12.9 | 14 | 15.2 | 16.4 | 17.7 | 19 | 20.5 |  | 2.5 | 5.3 | 8 | 10.7 | 13.4 | 16 | 18.8 |  | 0 | 0.6 | 1.1 | 1.7 | 2.3 | 2.8 | 3.4 |
|  | 70 | 12.5 | 13.5 | 14.6 | 15.7 | 17 | 18.3 | 19.9 |  | 1.5 | 4.4 | 7 | 9.7 | 12.5 | 15.1 | 18 |  | 0.1 | 0.7 | 1.2 | 1.8 | 2.4 | 2.9 | 3.5 |
|  | 80 | 12 | 13 | 14 | 15.1 | 16.3 | 17.7 | 19.3 |  | 0.9 | 3.6 | 6.2 | 8.8 | 11.4 | 14 | 16.7 |  | -0.3 | 0.5 | 1.3 | 2.1 | 2.9 | 3.7 | 4.5 |
|  | 90 | 11.5 | 12.5 | 13.4 | 14.5 | 15.7 | 17 | 18.6 |  | 0.6 | 3.1 | 5.5 | 7.9 | 10.3 | 12.6 | 15.1 |  | 0.1 | 0.7 | 1.4 | 2 | 2.6 | 3.3 | 3.9 |

Supplemental table 2. Descriptive models of participant characteristics in association with body composition status of the study population.

|  | FFMI | | | FMI | | | FFM to FM | | | BMI | | |
| --- | --- | --- | --- | --- | --- | --- | --- | --- | --- | --- | --- | --- |
|  | beta | SE | P value | beta | SE | P value | beta | SE | P value | beta | SE | P value |
| ***Men*** |  |  |  |  |  |  |  |  |  |  |  |  |
| Age | -0.04 | 0.01 | 0.000 | -0.01 | 0.01 | 0.447 | -0.01 | 0.01 | 0.277 | -0.05 | 0.02 | 0.004 |
| Kikuyu Tribe | -0.02 | 0.21 | 0.909 | 0.59 | 0.29 | 0.043 | -0.45 | 0.19 | 0.019 | 0.57 | 0.43 | 0.192 |
| Kalenjin Tribe | -0.51 | 0.23 | 0.027 | 0.00 | 0.33 | 1.000 | -0.24 | 0.22 | 0.265 | -0.49 | 0.49 | 0.311 |
| Urban | 0.81 | 0.16 | 0.000 | 1.44 | 0.23 | 0.000 | -0.57 | 0.15 | 0.000 | 2.27 | 0.34 | 0.000 |
| Primary Education | 0.39 | 0.19 | 0.043 | -0.13 | 0.27 | 0.626 | 0.14 | 0.18 | 0.425 | 0.22 | 0.41 | 0.591 |
| Secondary/College/Univ | 0.87 | 0.23 | 0.000 | 0.48 | 0.32 | 0.140 | -0.19 | 0.21 | 0.378 | 1.33 | 0.48 | 0.005 |
| Former smoker | -0.29 | 0.19 | 0.133 | -0.12 | 0.28 | 0.672 | 0.24 | 0.18 | 0.189 | -0.42 | 0.41 | 0.308 |
| Current smoker | -1.51 | 0.22 | 0.000 | -1.86 | 0.31 | 0.000 | 0.79 | 0.20 | 0.000 | -3.32 | 0.46 | 0.000 |
| Former alcohol | -0.09 | 0.18 | 0.618 | -0.10 | 0.25 | 0.697 | -0.22 | 0.16 | 0.182 | -0.14 | 0.37 | 0.695 |
| Current alcohol | -0.29 | 0.21 | 0.160 | -0.30 | 0.29 | 0.303 | -0.04 | 0.19 | 0.829 | -0.60 | 0.44 | 0.168 |
| Constant | 19.52 | 0.67 | 0.000 | 6.38 | 0.95 | 0.000 | 4.43 | 0.63 | 0.000 | 25.97 | 1.41 | 0.000 |
| ***Women*** |  |  |  |  |  |  |  |  |  |  |  |  |
| Age | -0.04 | 0.01 | 0.000 | -0.04 | 0.02 | 0.009 | 0.01 | 0.00 | 0.154 | -0.08 | 0.02 | 0.000 |
| Kikuyu Tribe | 0.09 | 0.21 | 0.650 | 0.64 | 0.45 | 0.152 | -0.19 | 0.11 | 0.089 | 0.75 | 0.61 | 0.215 |
| Kalenjin Tribe | -0.03 | 0.23 | 0.899 | 0.32 | 0.50 | 0.531 | -0.02 | 0.13 | 0.849 | 0.29 | 0.68 | 0.668 |
| Urban | 0.60 | 0.14 | 0.000 | 1.61 | 0.31 | 0.000 | -0.05 | 0.08 | 0.502 | 2.24 | 0.42 | 0.000 |
| Primary Education | 0.52 | 0.13 | 0.000 | 1.15 | 0.28 | 0.000 | -0.23 | 0.07 | 0.002 | 1.68 | 0.39 | 0.000 |
| Secondary/College/Univ | 0.79 | 0.20 | 0.000 | 2.20 | 0.44 | 0.000 | -0.42 | 0.11 | 0.000 | 3.05 | 0.60 | 0.000 |
| Former smoker | -0.80 | 1.04 | 0.440 | -0.04 | 2.23 | 0.984 | -0.16 | 0.57 | 0.782 | -0.81 | 3.03 | 0.790 |
| Current smoker | -0.87 | 0.81 | 0.283 | -1.51 | 1.73 | 0.384 | 0.91 | 0.44 | 0.039 | -2.33 | 2.36 | 0.323 |
| Former alcohol | -0.23 | 0.13 | 0.076 | -0.17 | 0.27 | 0.526 | -0.02 | 0.07 | 0.746 | -0.43 | 0.37 | 0.248 |
| Current alcohol | -0.74 | 0.25 | 0.004 | -1.64 | 0.54 | 0.003 | 0.39 | 0.14 | 0.005 | -2.44 | 0.74 | 0.001 |
| Constant | 18.32 | 0.54 | 0.000 | 11.28 | 1.16 | 0.000 | 1.80 | 0.29 | 0.000 | 29.61 | 1.58 | 0.000 |

Reference levels: Mixed tribe; rural; no education

The coefficient for age is the increase for 1 year.
